# Scrotal Abscess as Initial Presentation of Squamous Cell Carcinoma

**DOI:** 10.1155/2013/807346

**Published:** 2013-09-11

**Authors:** Kathy H. Huen, Paymon Nourparvar, John J. DeCaro, Mark D. Walsh, Muta M. Issa, Chad W. M. Ritenour

**Affiliations:** ^1^Department of Urology, Atlanta VA Medical Center and Emory University School of Medicine, 1365 Clifton Road, Atlanta, GA 30322, USA; ^2^Division of Plastic and Reconstructive Surgery, Atlanta VA Medical Center and Emory University School of Medicine, 1365 Clifton Road, Atlanta, GA 30322, USA

## Abstract

We report a case of scrotal squamous cell carcinoma in a 67-year-old man that presented as a recurrent nonhealing scrotal abscess. Radical scrotectomy and bilateral simple orchiectomy were performed. A pudendal thigh flap was used for wound closure. To our knowledge, this is the first report of its use after radical surgery for scrotal cancer. The clinical features, staging, and treatment of scrotal squamous cell carcinoma are reviewed. In this report, we highlight the importance of including scrotal cancer in the differential diagnosis when evaluating a scrotal abscess.

## 1. Introduction

Squamous cell carcinoma (SCC) of the scrotum is a rare neoplasm, with an incidence rate of 0.95/1 million [1]. In 1775, Pott reported greater scrotal cancer rates in chimney sweepers, the first description of an occupationally related cancer. It has subsequently been associated with exposure to tar, arsenic, paraffin, shale oil, petroleum wax, sun exposure, and human papillomavirus [2]. Currently, most cases are thought to result from poor hygiene and chronic inflammation [3]. SCC of the scrotum is most frequently diagnosed in the sixth and seventh decades. It typically presents as a solitary, painless, and slow-growing nodule. Ulceration may follow with an increase in lesion size, and the area can become infected [3].

In this report, we emphasize the importance of including scrotal cancer in the differential diagnosis when evaluating a scrotal abscess despite the lack of risk factors. We also describe the first reported use of a pudendal thigh flap for perineal wound closure after radical surgery for treatment of scrotal cancer.

## 2. Case Report

A 67-year-old man initially presented eight years ago for evaluation at an outside facility for a scrotal mass and purulent drainage. He was diagnosed with a scrotal abscess and incision and drainage was performed. The wound healed poorly, and the lesion recurred. In the subsequent years, he underwent scrotal exploration and two additional incisions and drainages for presumed recurrent abscesses. Twelve months prior to referral to our clinic, the scrotal lesion had been slowly enlarging and was draining foul-smelling, purulent discharge. He had lost 25 pounds in the preceding six months. He had no prior exposure history or occupational risk factors for scrotal cancer.

**Figure 1:**
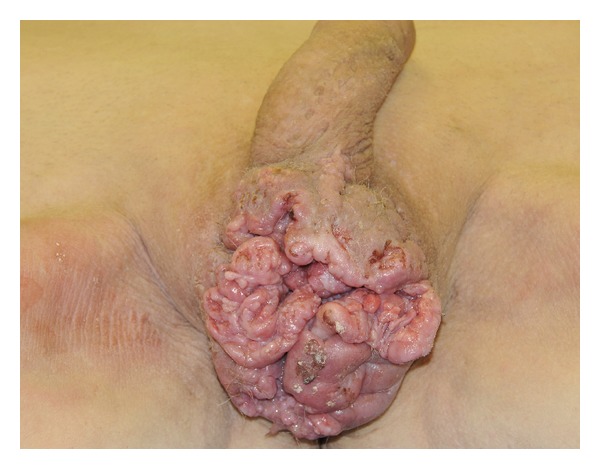
Late presentation of scrotal lesion, initially managed as scrotal abscess.

On examination, testes were palpable, but they were obscured by an irregular-appearing, ulcerating, fungating mass that distorted normal anatomy (Figure 1). Multiple firm, mobile, and enlarged inguinal lymph nodes were palpable bilaterally, with the largest measuring 2 cm in diameter. Laboratory investigations included white blood cell count of 11.9 × 10^9^/L (normal range, 4 × 10^9^ to 11 × 10^9^/L), and C-reactive protein was elevated at 433 nmol/L (normal range, 0.76 to 28.5 nmol/L). Other investigations including kidney function tests, liver function tests, lactate dehydrogenase, and urine examination were within normal limits.

**Figure 2:**
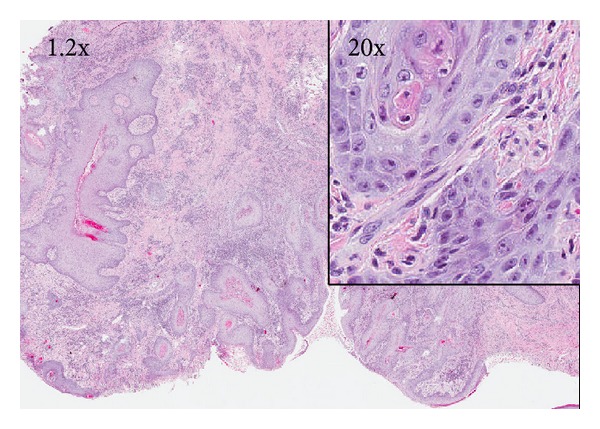
Hematoxylin-eosin stain (1.2x and 20x (right inset)) of scrotal lesion revealing well-differentiated squamous cell carcinoma with submucosal invasion and focal keratinization.

Three excisional biopsy specimens showed invasive well-differentiated SCC of the scrotum (Figure 2). Computed tomography (CT) revealed a large area of irregular skin nodularity and ulceration involving the scrotum, which abutted and was inseparable from both testicles, such that invasion could not be excluded. Enlarged bilateral inguinal nodes and mildly enlarged external iliac chain lymph nodes were also noted. There was no obvious evidence of distant metastatic disease on imaging.

Direct invasion of the testicles was difficult to exclude on clinical evaluation. After discussion with the patient, he decided to proceed with bilateral orchiectomy, with knowledge that the testes may not be involved, regardless of perioperative findings. Therefore, radical scrotectomy, bilateral simple orchiectomy, and reconstruction with pudendal thigh flap were planned. 

**Figure 3:**
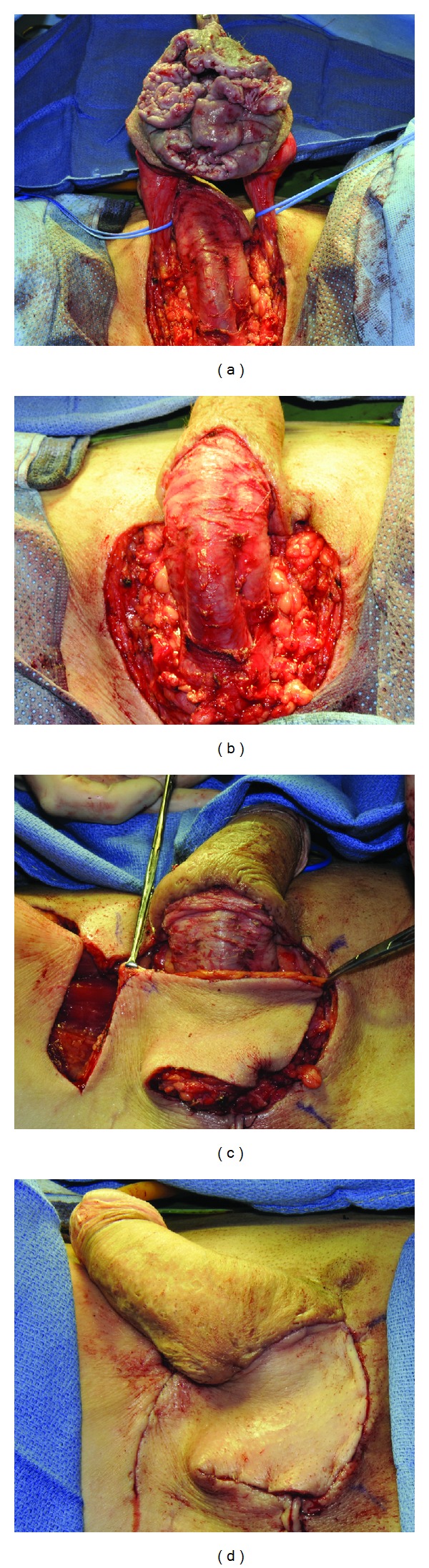
(a) Wide local excision of lesion and isolation of spermatic cords. (b) Completion of radical scrotectomy and bilateral simple orchiectomy. (c) Pudendal thigh flap mobilized superiorly for closure. (d) Completion of reconstruction with pudendal thigh flap.

After exclusion of urethral involvement with cystourethroscopy, wide local excision of the scrotum was performed. Surrounding subcutaneous tissue was excised. The spermatic cords were isolated and the testes were resected. A pudendal flap from the right thigh was used for perineal wound closure (Figure 3).

Histopathologic examination of the resected scrotum demonstrated well-differentiated squamous cell carcinoma extending to the deep dermal soft tissues. The tumor did not invade either the testicle or the spermatic cord. No angiolymphatic or perineural invasion was identified. All resected margins were negative for carcinoma.

**Figure 4:**
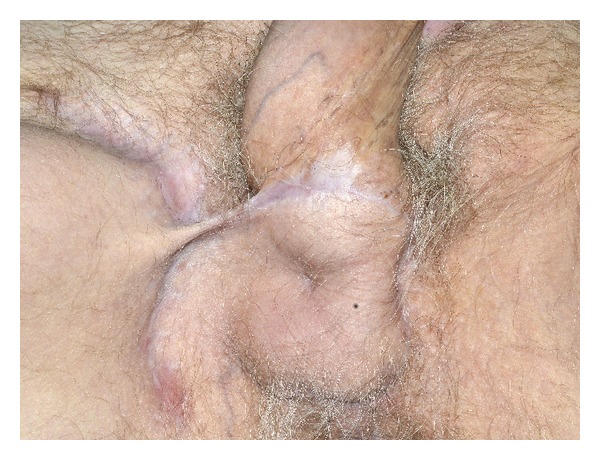
Perineal wound 5 months after radical scrotectomy, bilateral simple orchiectomy, and wound closure with pudendal thigh flap.

The patient made an uneventful recovery and was discharged on the fourth postoperative day with a course of antibiotics. At follow-up in two weeks, his perineal wound was healing well without signs of infection or palpable lymphadenopathy. Subsequent positron emission tomography-computed tomography (PET-CT) of the whole body showed resolved inguinal lymphadenopathy and no evidence of active cancer. At six-month follow-up, he had made a full recovery and reported full sensation in the perineal area (Figure 4). The patient will continue follow-up with urology, and consultations with medical and radiation oncology are scheduled to assess need for adjuvant therapy.

## 3. Discussion

Scrotal SCC is a rare malignancy. Patients typically present with a solitary, slow-growing, and painless skin lesion. It can be associated with ulceration or bleeding as the lesion enlarges in the later phases. Scrotal SCC is usually limited to one side of the scrotal sac, with predilection to the anterior inferior surface of the scrotum. Approximately 50% to 75% of patients have enlarged inguinal lymph nodes at time of initial presentation [4]. The mean delay to diagnosis in a case series of 14 patients was 22 months, with a range of 2 months to 10 years [5]. The delayed diagnosis of scrotal SCC in our patient serves as a grave reminder of unusual presentations of SCC and emphasizes the importance of including malignancy in the differential diagnosis when evaluating a scrotal abscess.

In 1983, Lowe modified the initial staging system proposed by Ray and Whitmore, which has now been widely accepted as the standard [6] (Table 1). An analysis of 766 patients showed that prognosis is strongly linked to stage but not to grade of tumor [5]—the median survival for localized, regional, and distant diseases was 115 months, 73 months, and 6 months, respectively [7].

Wide local excision is the principal treatment for scrotal SCC. Neither radiation therapy nor topical chemotherapy is proven to be effective [4]. The appropriateness of inguinal lymph node dissection at time of primary surgery remains controversial. Graves and Flo [2] propose bilateral radical groin dissection to remove micrometastases, while Ray and Whitmore [8] advocate withholding node dissection until there is clinical suspicion of nodal metastases. In our patient, we opted to assess for regional or distant nodal metastasis by PET-CT.

Split-thickness skin grafts are typically used for reconstruction after resection of extensive scrotal cancer. Use of pudendal thigh flap for scrotal and perineal reconstruction has been most frequently reported after tissue loss from Fournier's gangrene and trauma. This flap is sensate and has minimal donor-area morbidity, making it suitable for scrotal reconstruction. It has been proven to be reliable with good and rapid healing in most of the patients [9]. According to a pinned search in PubMed with MeSH terms “scrotum,” “neoplasms,” and “reconstructive surgical procedures,” this is the first use of the pudendal thigh flap after radical scrotectomy and bilateral simple orchiectomy for scrotal SCC.

## 4. Conclusion

Squamous cell carcinoma of the scrotum is a rare neoplasm. In patients who present with nonhealing recurrent scrotal abscesses, it is critical to include scrotal SCC in the differential diagnosis. Surgical treatment with radical scrotectomy and bilateral simple orchiectomy with the use of a pudendal thigh flap for wound closure achieves excellent recovery, good aesthetic results, and few complications.

## Figures and Tables

**Table 1:** Lowe's staging of scrotal SCC.

Stage	Description
A1	Disease localized to scrotum

A2	Locally extensive disease involving adjacent structures (penis, perineum, testis or cord, and pubic bone) by continuity but without evident metastasis

B	Superficial lymph node metastasis, resectable

C	Pelvic lymph node metastasis or any unresectable metastasis

D	Distant metastasis beyond regional nodes
